# Anti-diabetic effect of *Capparis spinosa *L. root extract in diabetic rats

**Published:** 2015

**Authors:** Mostafa Kazemian, Mansur Abad, Mohammad reza Haeri, Mansoor Ebrahimi, Reza Heidari

**Affiliations:** 1 *Department of Biology, Faculty of Science, University of Qom, Qom, Iran*; 2 *Department of Clinical Biochemistry, Faculty of Medicine, Qom University of Medical Sciences, Qom, Iran*; 3 *Booali Medical Research Center, Qom, Iran*; 4 *Anesthesiology Section, Qom University of Medical Sciences, Qom, Iran*

**Keywords:** *capparis spinosa*, *diabetes mellitus*, *insulin*, *glibenclamide*

## Abstract

**Objective:**

Diabetes mellitus is the most common metabolic disorders with severe impact on quality of life. Reducing serum glucose levels and normalization of serum lipid is of great clinical importance for treating diabetes. To our knowledge, there are not any evidences about the anti-diabetic action of *capparis spinosa* root. In the present study the effects of the* C. spinosa *root extract on diabetic metabolic disorders have been studied in experimental diabetes.

**Materials and Methods:**

Rats were divided into six groups: normal control (NC), diabetic control (DC), diabetic rats receiving 0.2, 0.4 g/kg of plant extract or 0.6 mg/kg glibenclamide (groups D0.2, D0.4 or DG respectively). A normal group of rats was also designed to receive 0.2 g/kg of plant extract (N0.2). Rats were rendered diabetic (streptozotocin 60 mg/kg, i.p.) and treated with 0.2, 0.4 g/ kg of plant extract or glibenclamide for four weeks. At the end of the experiment, blood was drawn through heart puncture under deep anesthesia. Weight was measured weekly, glucose levels were measured at the first and fourth week and lipid profiles, insulin and liver enzymes at the end of the study.

**Results:**

Glucose levels significantly decreased after treating with plant extract (p=0.003). However, insulin levels did not increase in any treating groups. Plant extract could significantly raise HDL and reduce levels of LDL and liver enzymes (ALT and ALP).

**Conclusion:**

These results showed that* C. spinosa *root extract could improve diabetic related metabolic derangement such as hyperglycemia, dyslipidemia, and elevated liver markers in an insulin-independent manner.

## Introduction

Diabetes mellitus is a major challenge for health care systems around the world (González-Villalpando et al., 2008 ▶). Type I diabetes is caused by immune system-mediated destruction of insulin-producing beta cells in the pancreatic islets (Tsai et al., 2008 ▶). Clinically, diabetes mellitus is one of the most important risk factors for cardiovascular diseases (Tripathi et al., 2006 ▶). Currently, more than 150 million people worldwide are suffering from this disease and it is expected that the number of them reaches 366 million in 2030 (Wild et al., 2004 ▶). The main goal of diabetes treatment is establishing of normal levels of blood glucose and preventing or delaying its metabolic complications (Nesto et al., 2001 ▶). Although insulin is the main remedy used in type I and in some cases in type II diabetes, there is a great need to find new drugs with minimal side effects (Grover et al., 2002 ▶ and Khan et al., 2003 ▶). Herbs, due to their ease of access and fewer side effects, have been the main cure for several diseases such as diabetes mellitus in ancient medicine (Grover et al., 2002 ▶). *Capparis spinosa’s *root have many valuable biochemical compounds such as flavonoids, saponins, tannins, pectin, essential oils, and particularly glycosinolate and glycosides (Khanfar et al., 2003 ▶; Matthaus and Ozcan, 2005 ▶; Sharaf, El-Ansari and Saleh, 2000 ▶ and Yang, Liu, Wang, 2008 ▶). Studies have shown that within two weeks of oral administration, *C. spinosa *aqueous extract decreases cholesterol and triglyceride levels in streptozotocin-induced diabetic rats (Eddouks, Lemhadri, Michel, 2005 ▶). In another study, fruit aqueous extract of the plant in combination with caraway (*Carum carvi*) showed hypoglycemic effect in diabetic animal models (Eddouks, Lemhardi and Michel, 2004 ▶). Different parts of the plant (fruits, seeds, leaves, etc.) may have different active ingredients and hypoglycemic properties. In the present study, anti-diabetic effects of *C. spinosa’s *root hydroalcoholic extract was examined in streptozotocin-induced diabetic and healthy adult male rats.

## Materials and Methods

**Plant material and extraction procedure**

*C. spinosa’s* roots were collected in Qom (Iran) and identified and registered (#45632) by herbarium of the Department of Biology, University of Qom (Iran). Roots were dried at room temperature and then were ground and macerated (100 g/400 ml) in ethanol (70%) for 24 hours with shaking. The extract was passed through a filter paper and concentrated by gentle heating (40 °C). 

**Animals**

In this experimental study, 36 adult male Wistar rats (Pasteur Institute, Tehran, Iran) weighing 200-250 were used. Rats were housed in separate cages in an animal room with constant temperature 23±2 °C, 12 h light–dark cycle and a relative humidity of 40 to 60 percent. They also had an unrestricted access to water and standard chow (Chavdaneh, Isfahan, Iran). All animals were given the opportunity to adapt to the environmental conditions for two weeks before the induction of diabetes. The animals were treated in accordance with the standard guideline (National Research Council, Institute of Laboratory Animal Resources, 1996) and Animal Ethics Committee of the University of Medical Science, Qom, Iran. 

All of the rats were divided into six groups each of six, normal rats receiving distillated water that designed as normal control (NC), STZ-induced diabetic control (DC) receiving distilled water, STZ-induced diabetic rats receiving 0.2 g/ kg (group D0.2), STZ-induced diabetic rats receiving 0.4 g/ kg (group D0.4) of hydroalcoholic extract of *C. spinosa *root, normal rats that received root extract at a dose of 0.2 g/ kg (C0.2), and diabetic rats received 0.6 mg/ kg of glibenclamide as a reference drug (DG) (chemidarou Pharmaceuticals, Iran) (Asgary et al., 2012 ▶). All of the animals received treatments by gavage for 28 days. Blood glucose concentration was measured at the first and last week (fourth week) of the study period. Weight was measured weekly.

**Induction of diabetes**

Rats were rendered type I diabetic by intraperitoneal injection of streptozotocin (STZ) (Sigma-Aldrich, Germany) at a dose of 60 mg/kg (Samarghandian et al., 2012 ▶). Blood glucose concentration was measured by a glucometer (GlucoDr, Germany) on a drop of blood from the tail. Rats with blood glucose levels greater than 300 mg/dL were considered to be diabetic (Samarghandian et al., 2012 ▶).

**Blood sampling and biochemical analysis**

After 28 days of treatment, the animals were deeply anesthetized with ether and blood samples were drawn through heart puncture, centrifuged (1500 g for 10 min) and sera were separated and stored in the freezer until assay. Levels of triglycerides, cholesterol, LDL, HDL, and activity of liver enzymes alanine transaminase (ALT), aspartate transaminase (AST), and alkaline phosphatase (ALP) were measured using commercial kits (Biosystems, Spain). Insulin was assessed using rats insulin ELISA kit (Mercodia, Sweden). 

**Statistical analysis**

All raw data were analyzed using one-way ANOVA, student's paired t-test and Tukey's post-hoc test using SPSS (version 18) and expressed as mean ± SD. P<0.05 was considered to be statistically significant. 

## Results

**Figure1:**
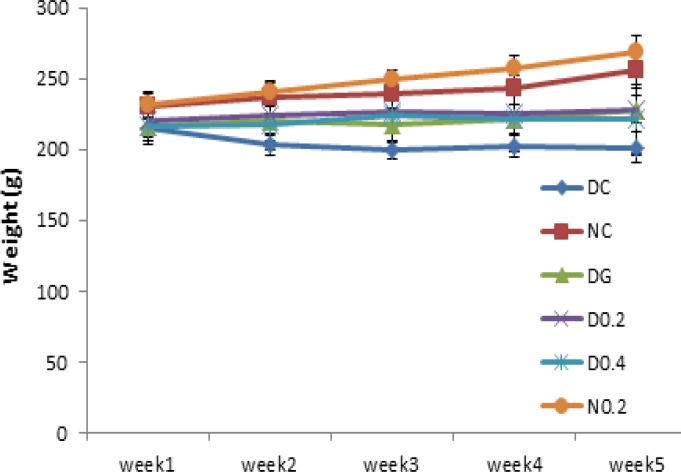
Body weight of the rats throughout the experiment (week 1 to 5). Data are expressed as mean±SD of measurements from 6 rats. Groups are labelled as follows: NC: normal control, DC: diabetic control, DG: diabetic rats treated with glybencelamide, D0.2 and D0.4: diabetic rats treated with 0.2 and 0.4 g/kg of hydro-alcoholic extract of the *C. spinosa *root, and N0.2: normal control treated with 0.2 g/kg plant extract

There was no significant difference in body weight and blood glucose levels between groups before induction of diabetes. After Five days of injection, diabetes symptoms including weight loss and polydipsia appeared. DC showed significant and continuous weight loss after induction of diabetes throughout the study in comparison with NC (p= 0.0001). D0.4 and DG showed a significant weight gain (p<0.05) compared to DC during the third week to the end of the study (Figure 1), while weight gain in D0.2 was not significant.

**Figure 2:**
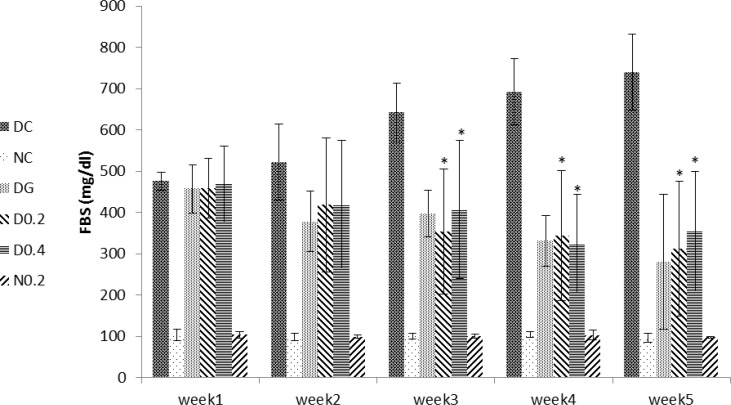
Levels of serum glucose through week 1 to 5. Data are expressed as mean±SD of measurements from 6 rats. Groups are labelled as follows: NC: normal control, DC: diabetic control, DG: diabetic rats treated with glybencelamide, D0.2 and D0.4: diabetic rats treated with 0.2 and 0.4 g/kg of hydroalcoholic extract of the *C. spinosa *root, and N0.2: normal control treated with 0.2 g/kg plant extract. *: p˂0.05 in comparison with DC

Serum glucose levels significantly increased in DC compared to NC after induction of diabetes (p= 0.0001) which persisted until the end of the study period (Figure 2). A significant decrease in blood glucose level was observed in D0.4 (p<0.05) and D0.2 (p=0.008) compared to DC that started from third week until end of the study (Figure 2). 

A significant and continuous reduction in blood glucose level was also observed in DG, compared to DC (p=0.001) (Figure 2). It is notable that there were no significant differences in serum glucose levels between D0.2, D0.4 and DG and between C0.2 and NC (Figure 2).

**Figure 3:**
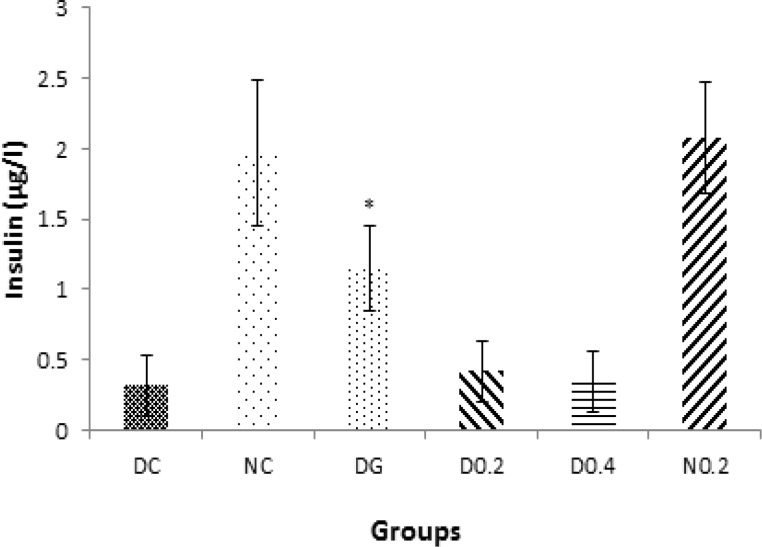
Levels of serum insulin through week 1 to 5. Data are expressed as mean±SD of measurements from 6 rats. Groups are labelled as follows: NC: normal control, DC: diabetic control, DG: diabetic rats treated with glybencelamide, D0.2 and D0.4: diabetic rats treated with 0.2 and 0.4 g/kg of hydro-alcoholic extract of the *C. spinosa *root, and N0.2: normal control treated with 0.2 g/kg plant extract. *: p˂0.05 in comparison with DC.

Insulin dropped sharply after induction of diabetes (p<0.005). Plant extract did not affect insulin levels in treated groups, but glibenclamide increased insulin levels in DG (p<0.01, Figure 3).

**Figure 4:**
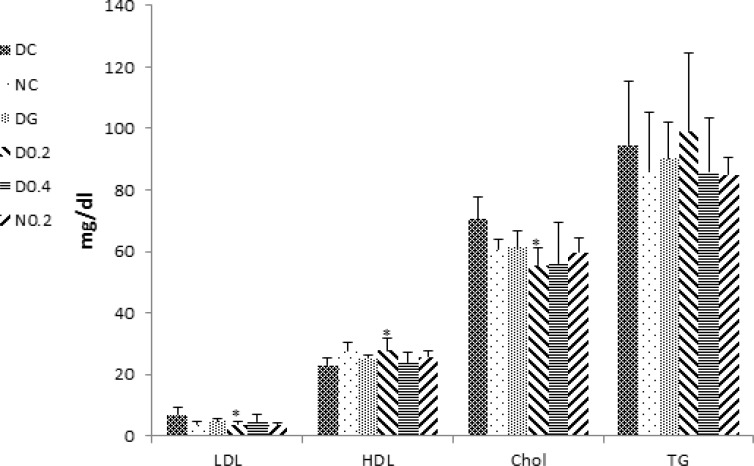
Levels of serum lipids, LDL, HDL, cholesterol, and triacylglycerol. Data are expressed as mean±SD of measurements from 6 rats. Groups are labelled as follows: NC: normal control, DC: diabetic control, DG: diabetic rats treated with glybencelamide, D0.2 and D0.4: diabetic rats treated with 0.2 and 0.4 g/kg of hydro-alcoholic extract of the *C. spinosa *root and N0.2: normal control treated with 0.2 g/kg plant extract. *: p˂0.05 in comparison with DC

After induction of diabetes, cholesterol significantly increased in DC (p<0.05) and decreased in D0.2, DG, and D0.4 as compared to DC (p=0.001, p<0.01 and p<0.05 respectively) (Figure 4). 

However, no significant changes in triglyceride levels were observed in any treated groups (Figure 4). LDL and HDL cholesterol levels tended to normal levels in D0.2 as compared to the DC (p<0.05) (Figure 4).

**Figure 5:**
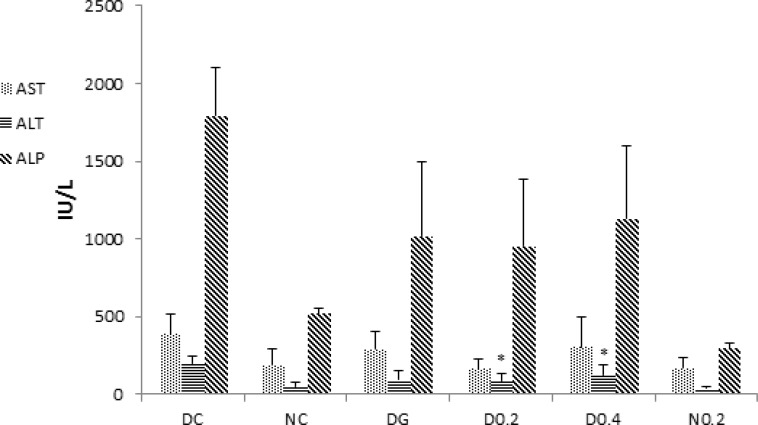
Levels of serum liver enzymes aspartate aminotransferase (AST), alanine aminotransferase (ALT), and alkaline phosphatase (ALP). Data are expressed as mean±SD of measurements from 6 rats. Groups are labelled as follows: NC: normal control, DC: diabetic control, DG: diabetic rats treated with glybencelamide, D0.2 and D0.4: diabetic rats treated with 0.2 and 0.4 g/kg of hydro-alcoholic extract of the *C. spinosa *root, and N0.2: normal control treated with 0.2 g/kg plant extract. *: p˂0.05 in comparison with DC

In the case of liver enzymes, induction of diabetes led to significant increases in the serum activities of AST (p <0.05), ALT (p=0.001), and ALP (p=0.0001) (Figure 5). ALT and ALP activity showed a significant reduction in D0.2 and D0.4 (p<0.05 for both) but AST reduced only in D0.2 (p<0.05). Interestingly, normal healthy rats treated with 0.2 g/kg of the extract had serum ALP activity significantly lower than normal controls (p=0.0001) (Figure 5).

## Discussion

Diabetes mellitus is an endocrine disorder that is characterized by hyperglycemia (Brownlee, 2001 ▶) and some forms of dyslipidemia such as hypertriglyceridemia and decrement in HDL-C levels (Tripathi and Srivastava, 2006 ▶). Some of the currently used effective medications are glibenclamide, metformin (Inzuchi et al., 1998 ▶), alpha-glucosidase inhibitors (Scheen, 1997 ▶), and troglitezone (Sparano and Seaton, 1998 ▶). However, synthetic hypoglycemic agents, have some adverse effects and not always are able to keep blood sugar in the normal range (Dey, 2002 ▶). Over the centuries, plants due to ease of access and in some cases fewer side effects, have enjoyed a special place for the treatment of diseases (Grover, 2002 ▶). In the present study, the anti-diabetic effects of *C. spinosa *root extract was studied. The results show that plant extract had a valuable hypoglycemic effect and significantly could decrease blood glucose levels to the levels seen in glibenclamide treated group (p<0.005) but unlike glibenclamide, had no effect on the serum levels of insulin (comparison of insulin levels in D0.4, D0.2, and DG in Figure 3). This clearly shows that hypoglycemic effect of the plant extract is insulin-independent. As shown in Figure 1, plant extract had no hypoglycemic effect in normal rats, indicating no risk of hypoglycemic shock, an ideal property for an anti-diabetic drug.* C. spinosa *contains quercetin (Sharaf et al., 2000 ▶) that is known to reduce glucose levels in streptozotocin-induced diabetic rats but not in normal rats as seen in the present study (Vessal et al., 2003 ▶). In addition to reducing blood glucose, an insulin-mimetic agent should be able to normalize dyslipidemia. Our results showed that* C. spinosa *extract at dose of 0.2 g/kg was able to cause a significant decrease in cholesterol levels compared to diabetic controls (p<0.01). *C. spinosa *is rich in phytosterols (Sharaf et al., 2000 ▶) which are known to reduce cholesterol levels through different mechanisms (Yamamoto, 2006 ▶ and Matsuyama, 2007 ▶). Induction of diabetes also caused a significant increase in LDL-C and a reduction in HDL-C levels (Figure 4). However, treating diabetic rats with the extract (0.2 g/kg) significantly decreased LDL-C and increased HDL-C levels (p<0.05) while glibenclamide could not significantly affect LDL and HDL levels (p> 0.05 in comparison to DC). Quercetin and morin reduce serum cholesterol levels (Fabiane et al., 2001 ▶) possibly through increasing LDL receptors (Moon et al., 2012 ▶). The ability of the plant extract to reduce LDL may be attributed to quercetin and morin content of *Capparis spinosa*. The plant extract decreased ALT, AST, and ALP in the diabetic group. Treating rats with 0.4 g/kg of crude plant extract reduced ALT and ALP activity as much as glibenclamide (Figure 5). Moreover, normal rats that received the extract did not show any increase in serum liver enzyme activities. These results show that the plant extract does not have a toxic effect on the liver and seems to be hepatoprotective. Body weight loss is another major symptom of diabetes (Samarghandian et al., 2012 ▶). Weight reduction (compared to NC) started following induction of diabetes and continued until the end of the study. However, treating diabetic rats with the extract (0.4 g/kg) caused a significant weight gain in comparison to DC. This indicates that *C. spinosa *root extract reduced blood glucose levels not through reducing appetite and food intake. The interesting thing seen in our study was the weakness and the perturbation of diabetic animals, which not observed in normal or treated animals. This shows that metabolic and psychological characters of diabetic animals tend to normal condition after treatment. In summary, our data showed that *C. spinosa *could improve serum glucose, lipids, and liver biomarkers without any increase in insulin secretion or appetite reduction.

In summary, the results of this study showed hypoglycemic and hypolipidemic effects of oral administration of hydroalcoholic root extract of *Capparis spinosa*, in experimental models of diabetes mellitus in rats and beneficial changes in serum levels of liver enzymes. Any change in insulin levels after one month of treatment suggests an insulin-independent hypoglycemic mode of action of the extract. However, further investigation is needed to identify its exact mechanism of action before introducing as a new anti-diabetic medication.
